# Man and His Enemies

**Published:** 1889-05-11

**Authors:** 


### The Doctor's Pulpit.

### MAN AND HIS ENEMIES.

### The Wager of Battle.

There comes a time in practically every man's history when he finds out that he is a soldier of fortune. People talk of "self-made" men—using the words in a strictly limited sense. The truth is that no human creature is ever a man at all unless he is self-made. Carlyle tells us that nobody ever took the trouble to flatter the boy Martin Luther, because he was too poor and powerless to be worth flattering for anything that could be got out of him, or any use that could be made of him by the flatterer. And so young Luther, from a very early age, was made to see all things around him exactly as they were in their baldest literalness. That which happened to Luther always, happens to every human being at some time or other and in some form. No man, no king nor queen, need hope to escape it. If fellow-men flatter, cold and heat do not, a storm at sea does not, sickness does not, death does not. Sooner or later every man, and woman, and child is brought face to face with stern and inflexible reality.

Youth often contrives to evade realities that are unpleasant, or to get someone else to meet them. Enemies that are too strong can be run away from. A college examination may be shirked or postponed for a year; a pressing debt can wait for payment, or the account can be sent with a penitent letter to a father who remembers his own early days. But the best hunter, sooner or later, comes to a 10ft. wall which he cannot even attempt to clear; the wariest debtor meets with one tough creditor whose Shylockian instinct insists upon his pound of flesh—that and nothing else. "Nae man can tether time nor tide," wrote Burns, and no man, whatever be the ten thousand horse-power of his unflinching determination, can always get his will carried into effect. What a blessing both to him and to others that he cannot! Carlyle admired will and effectiveness to an absolutely fanatical extent. That was one of the main sources of his weakness in judgment and conduct. The battle of life has to be fought, and is unavoidable, but the weapons it has to be fought with, the spirit which is to guide the combatant, the object to be fought for, and the kind of victory to be won, these are all to a large extent within the discretion of every individual soldier.

The body fashions itself more or less after the intents of the mind. Just as a man's character gradually stamps itself upon his face, so, literally, does his habitual conduct impress itself upon each organ and tissue of his body. In order to perfect health, then, it is clear that we must begin in the region of the intellect. Youth, we suppose to be past; the wild oats are sown, and perhaps reaped; it has dawned upon us at last that life is not a stage play: it is a contest to the death in a variety of ways. We have probably found out that we have a liver that can be troublesome, a stomach that knows how to rebel, a heart that "worries" at inconvenient times, lungs that insist upon a certain amount of care, a brain that, like the tides of the sea, refuses to rise one single inch higher in effort, though all the navies in the world are stranded on the beach. The enemies of childhood are to a large extent hidden from the child, and are mostly encountered by parents, guardians, teachers, and others. But the enemies of manhood and womanhood show themselves boldly and plainly, and must be faced consciously. The brain of man is his commanding officer. On it the responsibility ultimately rests; its capacity or incapacity determines the success or failure of life's campaign.

What does a man propose to himself as his great aim in life? Many a man proposes nothing at all to himself. He does not seem to have the wit to look one single day ahead. Such a man proclaims himself a beggar's old shoe, which lies by the roadside for every chance traveller to kick at. If the "whips and scorns of time" are hard upon him, who is to be thanked for it? Himself only! If his carelessness lands him in an unsanitary home, if his stupidity saddles him with an incompetent medical attendant, if his idleness and inattention lead him into financial difficulties, he can blame nobody but himself. He has deliberately labelled himself a heedless fool, and everybody takes him at his own valuation. Every man of brains makes some kind of life-purpose for himself. He sets a goal before his eyes and "goes for it." The goal may be worth striving for or it may not. The way to it may be compatible with health and happiness or it may not. These are points for the man himself to determine. What principally concerns us here is the health question: health in its broadest sense, including physical vigour and mental satisfaction.

A man who cannot swim may jump into ten feet of water. In that case it requires neither a prophet nor a philosopher to tell us he will be drowned. Similarly a man may say, "I think myself fitted for a certain post and position in life, but the position is a long way off, the journey is beset with difficulties and dangers, and my travelling resources are of the most limited kind. Shall I venture?" It very often happens that the position to be won fills the mind to the complete exclusion of the dangers of the way and the poverty of the traveller's resources. Without sufficiently counting the cost, he boldly commits himself to the expedition. Ought he to be surprised if he finds himself in no long time in swamps and dismal forests, surrounded by enemies and dangers he never reckoned upon? Of course, if he is a brave man, he will fight his way out of the dangers. If possible, he will still push forward, and win the goal at all hazards. But it may not be possible. The physical strength may fail; the brain may be unequal to the occasions that arise. If this should prove to be the case, the man is not an unconquerable hero, but an invincible fool, if he does not with all speed reconsider his plans and resolve upon a fresh start. Not all men of great intellectual capacity and force of character are fitted to clear their way through difficulties to a kingdom, and then to reign successfully. They might have reigned if the kingdom had come to them by inheritance; but the labour of conquering it has more than exhausted the physiological resources which Nature supplied them with at the outset. A wise man will do what he can: he will not attempt what he sees to be clearly impossible. "If it be difficult, it is done," said the Frenchman; "if it be impossible, it shall be done." That sounds heroic; but it has no other heroism than that which is in the sound. As a practical rule of conduct it is mere nonsense, and French nonsense.

"But a man must have a definite purpose in life, and a plan of campaign." Undoubtedly. He must have, not one purpose, but two; the one near and the other remote; the one for the day, the other for the lifetime. The day's purpose must be entirely practical and possible, and is to be carried out at all risks. The life purpose serves the end of a general indicator. It is to the life what a guiding star is to the mariner. It is to be steadily aimed at if it be worthy ; but if it be never fully attained, the life is still not necessarily a failure. But the daily purpose—the practical business of the moment—that must be done from day to day, or life can never come to any other end but overwhelming disaster. Now, the near purpose, the business of life from day to day, should always be so carried on as to be compatible with perfect health both of body and mind. That is an absolute condition of ultimate and final success. Every day's operations should pay its own physiological expenses, and whenever possible should leave a small balance to be placed to a reserve fund. If it be otherwise ; if the day's proceedings more than exhaust the day's physiological income, if capital be persistently drawn upon, what is and must be the inevitable result ? Is it not true that the man who is making creditors he can never hope to pay is creating hungry wolves who will one day rush upon him in an overwhelming pack and devour him ? What does it matter whether the wolves be financial or physiological ? A wolf is a wolf, and he must and will have food. The natural difficulties of any man's life are quite sufficient to test his capacity without the unnecessary creation of new ones. The natural enemies that beset his pathway at every stage are quite able to compass his destruction if he be not watchful and capable. The life-work undoubtedly demands effort of the most strenuous kind, and many a man thinks that if he put forth effort enough he ought to be successful, and he ought to be happy. But mere effort and strenuousness never were and never will be sufficient of themselves. Sense is imperatively demanded. The lion may conquer by hunger and rush, the man never. Nature will not give to man her highest rewards except on the condition of the highest use of a capable intellect. Of all the thousand enemies that lie ambushed along the journey of human life there is not one that does not shrink and cower before a clear intellect, a potent will, and an honest intent. The great and ultimate life-purpose with which any particular man sets out may be foolish, or it may be in itself impossible for him, and therefore may never be accomplished ; but the purpose of doing the work of the day wisely, well, and so that each day's resource shall be equal to each day's demand—that is, as a rule, possible for every man.

